# Risk factors for the occurrence of multidrug-resistant tuberculosis among patients undergoing multidrug-resistant tuberculosis treatment in East Shoa, Ethiopia

**DOI:** 10.1186/s12889-018-5371-3

**Published:** 2018-04-02

**Authors:** Fanta Desissa, Tilaye Workineh, Takele Beyene

**Affiliations:** 1 Department of Microbiology, Immunology and Veterinary Public Health, College of Veterinary Medicine and Agriculture, Addis Ababa University, P. O. Box 34, Bishoftu, Ethiopia; 2 Adama Hospital Medical College, P. O Box 84, Adama, Ethiopia; 3 Department of Biomedical Science, College of Veterinary Medicine and Agriculture, Addis Ababa University, P.O. Box 34, Bishoftu, Ethiopia

**Keywords:** MDR-TB, TB, Risk factors, Anti-TB drugs

## Abstract

**Background:**

Multidrug-resistant tuberculosis (MDR-TB) is resistant to the two main first-line anti-tuberculosis drugs: rifampicin and isoniazid. It is a major threat to public health worldwide. The objective of this study was to assess the potential risk factors for multidrug-resistant tuberculosis among patients undergoing MDR-TB treatment at two community hospitals in Ethiopia.

**Methods:**

A case-control study design was conducted from February 1, 2016, to April 29, 2016. TB-positive patients with MDR-TB and non-MDR-TB were considered as cases and controls, respectively. A total of 219 study participants were included in the study. An interviewer-administered structured questionnaire was used to collect primary data from the patients, and a checklist was used to collect data from the clinical records. Bivariate and multivariate logistic regression analyses were used to assess the potential risk factors for the occurrence of MDR-TB.

**Results:**

The odds of developing MDR-TB were higher in patients previously treated with anti-TB drugs (odds ratio [OR] = 6.1, 95%CI: 2.92–12.62, *P* < 0.001), those with a history of contact with known TB patients (OR = 2.1, 95%CI: 1.04–4.43, *P* < 0.001), those living in a rural setting (OR = 5.6, 95%CI: 2.14–14.46, *P* = 0.001), those with a history of alcohol consumption (OR = 4.3, 95%CI: 2.29–10.49, *P* < 0.001) and those without a job (OR = 2.4, 95%CI: 1.06–5.42, *P* = 0.001).

**Conclusions:**

The study revealed that contact with known TB patients, previous TB treatment, residence area, lack of a job, and alcohol consumption were potential risk factors for the occurrence of MDR-TB. Enhancing public health education, intensifying directly observed therapy programmes for all TB patients and designing control strategies are recommended.

## Background

Tuberculosis (TB) is an infectious disease caused by the bacillus *Mycobacterium tuberculosis*. Typically, it affects the lungs and other organs [1]. The disease is spread through the air when people who are sick with pulmonary TB expel the bacteria. Only a small proportion of people, approximately 10%, infected with *M. tuberculosis* will develop clinical TB during their lifetime, but this probability is much higher among immunocompromised individuals, such as those infected with HIV [2].

Tuberculosis is one of the world’s deadliest communicable diseases with most cases in Asia and Africa, including Ethiopia [3–5]. The emergence of multidrug-resistant TB (MDR-TB) is a challenge for the global control and prevention of the disease [6]. Multidrug-resistant tuberculosis is a type of TB that is resistant to at least the two main first-line anti-TB drugs, namely, rifampicin and isoniazid. People become infected with MDR-TB either when they are exposed to a resistant strain or when improper treatment leads to selection of a resistant strain [7]. Annually, approximately 3.3% of new TB patients and approximately 20% of previously treated patients become infected with MDR-TB, leading to the deaths of 190,000 individuals [1].

Ethiopia is one of the countries with the highest MDR-TB burdens, with 2.3% of newly confirmed TB patients affected and 17.8% of previously treated TB patients affected [1, 8, 9]. There is no citable information on the regional prevalence of MDR-TB among newly confirmed and previously treated TB patients, particularly in the Oromia region. However, the first population-based attempt to measure the prevalence of primary and secondary MDR-TB showed a prevalence of MDR-TB of 2.4% and 14.3% among newly confirmed and previously treated TB patients, respectively [10]. The rate of MDR-TB detection was estimated to be 33% among patients with presumptive MDR-TB, which is high compared to other parts of the country [11]. In the region, treatment centre-based management of MDR-TB has been implemented at different hospitals, including Adama Hospital and Bishoftu Hospital. Several factors have been connected to the development of MDR-TB [8, 12]. Addressing the underlying risk factors is one of the five principal pathways for preventing drug-resistant TB [3]. However, there is little citable and comprehensive evidence regarding the potential risk factors that are responsible for the development of MDR-TB in patients undergoing anti-TB treatment in the studied areas. Therefore, the objective of this study was to assess the risk factors associated with MDR-TB among MDR-TB patients following anti-TB treatments at Bishoftu Hospital and Adama Hospital.

## Methods

The study was conducted at Bishoftu Hospital and Adama Hospital between February 1, 2016 and April 29, 2016. These towns are located in the East Shoa Zone, Oromia Regional State, Ethiopia, which is southeast of Addis Ababa, the capital city of Ethiopia. The total populations in Bishoftu and Adama are 100,114 and 422,490, respectively [13]. A case-control study design was employed to determine the risk factors for MDR-TB. The source population included all confirmed MDR-TB patients (cases) and non-MDR-TB patients (controls) at Adama Hospital and Bishoftu Hospital undergoing anti-TB treatment during the study period. The study population included all the cases and controls accessible for sampling during the study period.

The inclusion criteria were MDR-TB and non-MDR-TB patients who were under treatment follow-up during the study period. MDR-TB patients were confirmed by culture and sensitivity testing. Non-MDR TB patients were either newly or previously treated TB patients who had smear microscopy that was positive for acid fast bacilli (AFB) or had clinical and/or radiological evidence of disease. Drug-sensitive TB was indicated by smear negative microscopy at the end of the intensive phase of treatment and at the 5th or 6th month of the continuation phase of TB treatment. Patients with non-confirmed MDR-TB or extensively drug-resistant tuberculosis (XDR-TB) were excluded.

A formula from Fleiss’ statistical methods for rates and proportions [14] was used to calculate the sample size with Open Epi version 7 statistical packages for Windows based on the following parameters: an estimated exposure of known TB contact for controls (11.3%) [15], a marginal error of 5%, an estimated odds ratio of 2.8, 80% power, a 95% confidence limit and a 1:2 ratio of cases to controls. Accordingly, the minimum required sample size was 215. All the MDR-TB patients who were registered and undergoing treatment at both hospitals (13 at Bishoftu Hospital and 102 at Adama Hospital) were targeted for selection due to the small number of cases. The controls were selected from the TB clinic patient registration book. Twenty-four TB patients were selected using a systematic random sampling method from 80 TB patients who were undergoing anti-TB treatment at Bishoftu Hospital, and 122 TB patients were sampled from 135 TB patients at Adama Hospital. Accordingly, a total of 219 participants including 73 cases (12 from Bishoftu Hospital and 61 from Adama Hospital) and 146 controls (24 from Bishoftu Hospital and 122 from Adama Hospital) were included in the study.

A pre-tested structured questionnaire was used to collect information from the study participants through a direct interview by researchers. A checklist was used to collect data from the clinical records. Training was provided to the individuals who were involved in the data collection process before the beginning of data collection. At the end of each interview, the principal investigator crosschecked the responses to the questionnaire by assessing the uniformity of the responses to multiple questions on the same topic to ensure completeness and data accuracy. Ethical clearance was obtained from the Oromia Health Bureau (Ref no. BEEFO/1–8/1239). A statement about the purpose of the study was read and explained to each study participant. Only those who gave verbal consent to participate in the study were included in the study.

The data were entered into an Excel spreadsheet and exported into SPSS statistical software for analysis. The data were assessed for completeness and consistency by calculating the frequencies of each variable. Bivariate and multivariate logistic regression analyses were carried out to test for the presence of an association between the dependent variable (MDR-TB) and each independent variable. Variables with a *P*-value less of than 0.1 in the bivariate analysis were included in the multivariate logistic regression model. For logistic regression, a step-wise forward entry method was used to determine the final model. A *P*-value < 0.05 in the multivariate logistic regression model was the cut-off point for statistical significance.

## Results

A total of 219 study participants including 73 cases and 146 controls were included in this study. Among the participants, there were relatively more females than males in both the case (54.8%) and control (58.2%) groups. The mean age of the study participants in the case and control groups was 32.69 (SD = 14.89) and 29.91 (SD = 12.57) years, respectively.

The univariate analysis showed that the lack of a job, living in a rural setting, a history of imprisonment, marital status, a history of alcohol consumption and cigarette smoking, the site or type of TB, previous TB treatment, a positive smear and a history of contact with a known TB patient were statistically associated with the development of MDR-TB (*P* < 0.05) (Table 1). However, upon conducting the multivariate logistic regression analysis, only a history of contact with a known TB patient (OR = 2.1, 95%CI: 1.04–4.43, *P* < 0.001), previous anti-TB treatment (OR = 6.1, 95%CI: 2.92–12.62, *P* < 0.001), living in a rural setting, the lack of a job (OR = 2.4, 95%CI: 1.06–5.42, *P* = 0.001) and a history of alcohol consumption (OR = 4.3, 95% CI: 2.29–10.49, *P* < 0.001) were statistically associated with the occurrence of MDR-TB in the study areas (Table 2).

**Table 1:** Univariate analysis showing the socio-demographic, behavioural and clinical factors associated with multidrug-resistant TB in patients undergoing anti-TB treatment at Adama Hospital and Bishoftu Hospital in the East Shoa Zone, Oromia

Variables	Case N (%)	Control N (%)	Crude OR(95%CI)	*P*-value
Age (completed years)				
< 25	23(31.5)	65(44.5)	1	
26–45	39(53.4)	62(42.5)	1.8(0.96–3.31)	0.700
46–87	11(15.1)	19(13)	1.6(0.68–3.95)	0.274
Sex				
Female	40 (54.8)	85(58.2)	0.9 (0.49–1.53)	0.629
Male	33 (45.2)	61(41.8)	1	
Ethnicity				
Amhara	19(26)	37(25.3)	1	
Oromo	48(65.8)	95(65.1)	0.9(0.51–1.89)	0.961
Others	6(8.2)	14(9.6)	0.84(0.28–2.52)	0.748
Education				
Illiterate	18(24.6)	35(24)	1.1(0.54–2.18)	0.814
Primary	20(27.4)	37(25.3)	1.1(0.58–2.25)	0.699
> Secondary	35(47.9)	74(50.7)	1	
Religion				
Muslim	16(21.9)	32(21.9)	1	
Orthodox	47(64.4)	83(56.8)	1.1(0.56–2.28)	0.727
Protestant	7(9.6)	31(21.2)	0.5(0.16–1.25)	0.127
Others	4(4.1)	0(0)	–	–
Residence				
Rural	23(31.5)	11(7.5)	5.6 (2.56–12.42)	0.000
Urban	50(68.5)	135 (92.5)	1	
Marital status				
Single	39(39.7)	82(56.2)	1	
Married	32(43.8)	58(39.7)	1.6(0.86–2.86)	0.149
Divorced	12(16.4)	6(4.1)	5.7(1.95–16.45)	0.010
Job status				
No	27(35.1)	25(16.9)	2.8(1.49–5.39)	0.001
Yes	46(64.9)	121(83.1)	1	
History of imprisonment				
Yes	12(16.4)	12(8.2)	2.2(0.93–5.17)	0.071
No	61(83.6)	134(91.8)	1	
Alcohol consumption				
Yes	40(54.8)	25(17.1)	5.9(3.12–11.02)	0.000
No	33(45.2)	121(82.9)	1	
History of smoking				
Yes	11(15.1)	10(6.8)	2.4(0.97–5.98)	0.057
No	62(84.9)	136(93.2)	1	
History of Chewing chat				
Yes	21(28.8)	29(19.9)	1.63(0.85–3.12)	0.141
No	52(71.2)	117(80.1)	1	
Site of TB				
Pulmonary TB	54(74)	83(56.8)	2.2 (1.16–3.99)	0.015
Extra Pulmonary TB	19(26)	63(43.2)	1	
Positive Smear				
No	18(24.7)	19(13)	1	
Yes	55(75.3)	127(87)	0.5(0.22–0.94)	0.033
Previous TB treatment				
No	25(34.2)	116(79.5)	1	
Yes	48(65.8)	30(20.5)	7.4 (3.66–13.92)	0.000
Contact with a known TB Patient				
No	28(38.4)	88(60.3)	1	
Yes	45(61.6)	58(39.7)	10.1(5.19–19.73)	0.000
HIV status				
Reactive	22(30.1)	45(30.8)	0.97 (0.53–1.78)	0.917
Non-reactive	51(69.9)	101(69.2)	1

**Table 2:** Multivariate analysis showing the risk factors for MDR-TB in patients undergoing anti-TB treatment at Adama Hospital and Bishoftu Hospital in the East Shoa Zone, Oromia

Variables	Case N (%)	Control N (%)	Crude OR (95%CI)	Adjusted OR(95%CI)	*P*-value
Previous treatment					
No	25 (34.2)	116 (79.5)	1	1	
yes	48 (65.8)	30 (20.5)	7.4 (3.66–13.92)	6.1 (2.92–12.62)	<.001
Contact with a known TB Patient					
No	28 (38.4)	88 (60.3)	1	1	
Yes	45 (61.6)	58 (39.7)	10.1 (5.19–19.73)	2.1 (1.04–4.43)	0.000
Residence					
Rural	26 (33.8)	6 (7.8)	5.6 (2.56–12.42)	5.6 (2.14–14.46)	0.001
Urban	51(66.2)	71(92.2)	1	1	
Job status					
No	27(35.1)	25 (16.9)	2.8 (1.49–5.39)	2.4 (1.06–5.42)	0.001
Yes	46 (64.9)	121(83.1)	1	1	
Alcohol consumption					
No	33 (45.2)	121 (82.9)	1	1	
Yes	40 (54.8)	25 (17.1)	5.9 (3.12–11.02)	4.3 (2.29–10.49)	0.000

## Discussion

A case-control study was conducted by recruiting 219 (73 cases and 146 controls) study participants to assess factors associated with developing MDR-TB. Factors such as contact with a known TB patient, previous anti-TB treatment, living in a rural setting, the lack of a job and a history of alcohol consumption were found to be statistically associated with the occurrence of MDR-TB. However, many factors related to socio-economic characteristics, personal behaviours and previous TB treatment-related factors were not statistically associated with MDR-TB in this study. The absence of a statistical association between these factors and MDR-TB in this study does not mean that they are not important factors affecting the occurrence of MDR-TB. These findings may be due to possible variations in the epidemiology of the disease and the socio-economic and health status of the community. Therefore, it is important to mention that the present findings do not mean that these factors are not related to MDR-TB. However, the authors opt to focus the following discussion on those variables that were found to be associated with the occurrence of MDR-TB in the studied areas.

MDR-TB was strongly associated with residence area; living in a rural area increased the occurrence of MDR-TB six times compared with living in an urban area. This was similar to a study conducted at the state level in Oromia and in the western part of Ethiopia, which found that rural residents were at higher risk of developing MDR-TB than urban dwellers [11, 16]. In contrast, residence area was not found to be associated with MDR-TB in a study conducted in Addis Ababa and China [17]. This difference may be due to differences in access to TB services, socio-economy and the level of awareness about adherence to first-line TB treatment, as rural communities have poor adherence to treatment that likely leads to MDR-TB. In the current study, marital status was not associated with the occurrence of MDR-TB. This is inconsistent with findings that have been reported elsewhere [11]. In contrast, in a study conducted in the Amhara region of the country, it was reported that being widowed was negatively associated with MDR-TB [18].

In this study, the lack of a job was associated with the occurrence of MDR-TB. This was in contrast with another study conducted in the USA, in which no significant difference was found in the occurrence of MDR-TB between the case and control groups [19]. The lack of job is connected with income and is an indicator of low socio-economic status. Several research reports indicated a high burden of MDR-TB among individuals of low socio-economic status [1, 8, 20]. The observed difference might be attributed to differences in income status.

The current study showed that alcohol consumption was associated with the occurrence of MDR-TB. Several reports, including one from the WHO, indicated that the use of alcohol increases the risk of developing MDR-TB due to poor adherence to treatment, impaired immune responses and an increased risk of adverse drug effects. As a result of these conditions, alcohol consumption was identified as an important population-level risk factor for MDR-TB [10, 16, 21]. The occurrence of MDR-TB was strongly associated with previous treatment with anti-TB drugs. This finding was consistent with several previous studies conducted elsewhere that indicated that previous exposure to TB treatment might be the most significant risk for MDR-TB [11, 16–18, 22–29]. The acquired drug resistance of *M. tuberculosis* to anti-TB drugs can occur when there is a history of incomplete or inappropriate TB treatment regimens lasting at least 1 month [30]. This may be because prior inadequate anti-TB treatment only suppresses the growth of susceptible bacilli and does not affect other resistant strains, leading to suitable conditions for the dominant multiplication of pre-existing drug-resistant mutants, which is a rise and fall phenomenon [15, 16, 31]. In a similar manner, MDR-TB patients in this study may have experienced similar conditions of previous inadequate treatment that led to the occurrence of MDR-TB.

In this study, cases were more likely to have had contact with TB patients than controls. The association between contact with known TB patient and MDR-TB was similarly observed in another very recent study conducted in the Oromia region [11]. Several other studies have also supported the hypothesis that contact with a known TB patient is linked with MDR-TB due to exposure to resistant TB resistant [24, 32, 33]. Either the transmission of MDR strains or the selection of single-drug-resistant strains may have contributed to the occurrence of MDR-TB among the cases [24].

The present study is not without limitations. First, the study participants were identified as MDR-TB and non MDR-TB patients only based on their clinical records from the respective TB and MDR-TB treatment centres. Second, due to the small number of MDR-TB patients undergoing anti-TB treatment at both hospitals, several factors, such as HIV status, which are known to be associated with the occurrence of MDR-TB, were not identified. Finally, as a case-control study, there might have been recall bias because some of the information collected was dependent on the recall capacity of the study participants.

## Conclusions

The present study revealed that previous exposure to anti-TB treatment, contact with known TB patients, living in a rural setting, a lack of a job and alcohol consumption were associated with an increased occurrence of MDR-TB. Enhancing public health education, intensifying the DOT programme for all TB patients, and regular monitoring of the standard treatment programme are recommended. Moreover, further studies should be conducted to clearly establish the epidemiological link and causal relationships between living in a rural setting, the lack of a job and alcohol consumption and the development of MDR-TB. Such findings would contribute to designing tailored control strategies aimed at identifying potential risk factors with particular emphasis on rural communities.

## Abbreviations

DOT: Directly observed therapy
MDR-TB: Multidrug-resistant tuberculosis
OR: Odds ratio
TB: Tuberculosis
XDR-TB: Extensively drug-resistant tuberculosis
